# The association between dietary inflammatory index with some cardio-metabolic risk indices among the patients with type 2 diabetes from Hoveyzeh cohort study: a cross-sectional study

**DOI:** 10.1186/s12902-024-01624-2

**Published:** 2024-06-19

**Authors:** Mehran Rahimlou, Amirhossein Ramezani Ahmadi, Bahman Cheraghian, Ghazal Baghdadi, Samira Sadat Ghalishourani, Shadi Nozarian, Seyed Jalal Hashemi, Zahra Rahimi, Nasrin Banaei Jahromi, Seyed Ahmad Hosseini

**Affiliations:** 1 Department of Nutrition, School of Public Health, Zanjan University of Medical Sciences, Zanjan, Iran; 2 Isfahan Endocrine and Metabolism Research Center, Isfahan University of Medical Sciences, Isfahan, Iran; 3 Department of Biostatistics and Epidemiology, School of Public Health, Ahvaz Jundishapur University of Medical Sciences, Ahvaz, Iran; 4 Department of Nutrition, School of Public Health, Iran University of Medical Science, Tehran, Iran; 5 Department of Physical Education and Sport Science, Science of Research Branch, Islamic Azad University, Tehran, Iran; 6 Department of Nutritional Sciences, School of Allied Medical Sciences, Ahvaz Jundishapur University of Medical Sciences, Ahvaz, Iran; 7 Alimentary Tract Research Center, Clinical Sciences Research Institute, School of Medicine, Ahvaz Jundishapur University of Medical Sciences, Ahvaz, Iran; 8 Department of Biostatistics and Epidemiology, School of Public Health, Ahvaz Jundishapur University of Medical Sciences, Ahvaz, Iran; 9 Nutrition and Metabolic Disease Research Center, Clinical Sciences Research Institute, Ahvaz Jundishapur University of Medical Sciences, Ahvaz, Iran

**Keywords:** Type 2 diabetes mellitus, Dietary inflammatory index, Lipid accumulation product, Body roundness index, Cardio-metabolic

## Abstract

**Background:**

The dietary inflammatory index (DII) serves as a tool to assess the inflammatory impact of an individual’s diet. This study aimed to investigate the association between DII and some cardio-metabolic risk indices among patients with T2DM.

**Methods:**

Data from the Hoveyzeh Cohort Study, encompassing 2045 adults with T2DM, were analyzed. DII scores were calculated based on food frequency questionnaires. Anthropometric measurements and biochemical tests were performed to assess cardio-metabolic risk factors.

**Results:**

Higher DII scores were positively associated with elevated triglyceride levels, triglyceride-glucose (TyG) index, lipid accumulation product (LAP), anthropometric indices including a body shape index (ABSI), body roundness index (BRI), body mass index (BMI), hip, waist circumferences (WC), and waist-to-height ratio (all P_trend_ < 0.05). Notably, no significant association was observed between DII and fasting blood sugar (FBS) levels (P_trend_ > 0.05). Additionally, dietary intake analysis revealed a negative correlation between DII scores and intake of fiber, fruits, vegetables, legumes, fish, seafood, dairy products, magnesium, and vitamins A, C, D, and E (all P_trend_ < 0.05). Conversely, higher DII scores were associated with increased consumption of red meat, processed meat, refined cereals, potatoes, and soft drinks (all P_trend_ < 0.05).

**Conclusion:**

This study underscores the critical link between dietary inflammation, assessed by the DII score, and a multitude of cardio-metabolic risk factors in patients with T2DM. Notably, while the study did not find a significant association between DII and fasting blood sugar levels, it identified robust associations with novel anthropometric and biochemical indices indicative of cardio-metabolic risk. These findings highlight the potential of dietary interventions as a cornerstone strategy for managing T2DM and mitigating its associated complications.

## Introduction

Chronic systemic inflammation, serving as the foundation for numerous chronic illnesses, is distinguished by heightened levels of inflammatory indicators [1, 2]. This persistent, low-grade inflammation throughout the body has been linked to various non-communicable diseases such as cancer, rheumatoid arthritis, and diabetes [3–5]. Diabetes Mellitus is a global health crisis affecting millions worldwide. The International Diabetes Federation (IDF)’s inaugural Diabetes Atlas in 2000 projected that 151 million adults globally had T2DM [6]. By 2019, this number had tripled to 463 million [7, 8]. This rising prevalence has resulted in a significant economic burden on healthcare systems. In 2007, the direct healthcare expenses associated with T2DM were estimated at US$232 billion, and by 2019, this figure had escalated to US$760 billion [9, 10]. Notably, 80% of individuals with T2DM reside in low- and middle-income countries, with a prevalence of around 14% reported in the Middle East, including Iran [11].

Dietary interventions have proven to be a cornerstone strategy in managing T2DM, demonstrably improving glycemic control and reducing cardiovascular risk [12–14]. Chronic inflammation is widely recognized as a key pathophysiological mechanism in the onset of T2DM [15]. The concept of dietary influence on inflammation is well-established, with certain dietary patterns promoting inflammation while others have anti-inflammatory properties. However, the precise impact of dietary manipulation on T2DM outcomes, particularly the role of inflammation, remains to be fully elucidated [16–20]. Therefore, a deeper understanding of the connections between diet, inflammation, and T2DM is crucial [21].

DII, a valuable tool developed by Cavicchia et al. [22] and refined by Shivappa et al. (2014), is designed to assess the overall inflammatory potential of a diet in diverse populations. Its utility lies in assessing the overall inflammatory impact of an individual’s diet, achievable through a 24-hour dietary record interview or the use of food frequency questionnaires (FFQs) [23]. DII score has been linked to markers of systemic inflammation and various chronic conditions associated with inflammation, including cardiovascular disease (CVD) [24], arthritis [25], and certain types of cancers [26].

Both obesity and overweight are well-established risk factors for T2DM, and T2DM itself can further worsen these conditions [27]. Anthropometry, employing simple body measurements, offers a cost-effective and accessible screening tool for cardiovascular disease, metabolic syndrome, and weight issues in individuals and populations [28]. However, while widely used in both clinical and epidemiological settings due to its simplicity, BMI’s limitation lies in its inability to differentiate between muscle and fat mass, leading to potential misclassification of overweight and obesity and underestimation of associated disease risks [29, 30]. This has driven the development of more precise anthropometric indices, such as ABSI, BRI and visceral adiposity index (VAI) to provide a more nuanced assessment [31, 32].

While the link between inflammatory biomarkers and inflammation-related chronic diseases is well-established, the association between the DII and T2DM related markers remains relatively underexplored. To address this gap, the present study investigated the association between DII scores and T2DM-related biochemical parameters and novel anthropometric indices in patients with T2DM from the Hoveyzeh Cohort Study.

## Methods

### Study design and sampling method

The Hoveyzeh Cohort Study (HCS) investigates non-communicable diseases (NCDs) in southwestern Iran [33]. This population-based cohort study recruited 10,009 adults aged 35–70 years between May 2016 and August 2018. Data collection and measurements followed standardized protocols ensuring consistency across the PERSIAN Cohort Study sites [33, 34]. In the current study, individuals were excluded if they had more than 10% unanswered items on their food frequency questionnaires (*n* = 122) or if their daily calorie consumption fell outside the range of 600 kcal to 5500 kcal (*n* = 13), as determined using the standard deviation method proposed by Rosner [35]. Moreover, individuals were excluded if they had incomplete biomarker data or lacked essential information on other covariates deemed significant (*n* = 46). A final sample of 2045 individuals were encompassed in our study.

Following written informed consent, a standardized, structured questionnaire was administered during in-person interviews to collect demographic information, dietary intake details via a food frequency questionnaire, and past medical history, tobacco use (defined as ever smoking one or more cigarettes daily for at least a month), alcohol consumption (defined as drinking alcohol at least once monthly), occupation, residential history, and other potential confounding variables.

This study was conducted in accordance with the ethical principles outlined in the Declaration of Helsinki. The study protocol and informed consent forms received thorough review and approval from the Ethics Committee of Ahvaz Jundishapur University of Medical Sciences (reference number: IR.AJUMS.REC.1398.760). All participants provided written informed consent after receiving a comprehensive explanation of the study’s objectives and methods.

### Dietary evaluation

Dietary intake was assessed using a validated semi-quantitative food frequency questionnaire (FFQ) previously employed in the Hoveyzeh Cohort Study [33]. This tool queried participants on their consumption frequency of 130 food items over the past year, with standard portion sizes indicated for each item. Recorded daily food consumption (grams) was then entered into Nutritionist IV software to calculate total energy and nutrient intake.

### Determination of DII

Dietary data from the validated FFQ was used to calculate DII scores for each participant. A comprehensive description of the DII method can be found elsewhere [23]. In summary, the corpus of literature pertaining to DII encompasses eligible publications spanning from 1950 to 2010, documenting associations between dietary components and various inflammatory markers such as Interleukin 1 beta (IL-1β), Interleukin 4 (IL-4), Interleukin 6 (IL-6), Interleukin 10 (IL-10), Tumor necrosis factor (TNF-α), and C-reactive protein (CRP). A total of 45 distinct food parameters associated with the six inflammatory biomarkers were identified in the literature review. Each parameter received a “food parameter-specific inflammatory effect score” by tallying the number of studies reporting pro-inflammatory, anti-inflammatory, and neutral effects on one or more of the six inflammatory markers. These scores were adjusted based on study design and the size of the literature for each food parameter’s inflammatory marker relationship. In prior analyses, there was a positive correlation between the DII and the circulating level of high-sensitivity C-reactive protein (hs-CRP) [36, 37]. In this study, DII scores for participants were computed by associating the dietary information with a global database, which furnished average intake values and standard deviations for each nutritional parameter. To adjust for the effect of energy, energy independent variables were calculated for these 45 nutrients. These values were subsequently used as factors to represent an individual’s exposure in relation to the ‘standard global mean,’ expressed as a z-score. This process involved subtracting the reported quantity from the ‘standard global mean’ and dividing the result by the standard deviation. As the data exhibited right-skewness, a typical observation in dietary data, we transformed these values into centered percentile scores. Each individual’s centered percentile score was then multiplied by the specific inflammatory effect score corresponding to the food parameter, resulting in an individualized food parameter-specific DII score. The cumulative sum of these food parameter-specific DII scores was subsequently calculated to derive the overall DII score for each participant in the study [23].

### Biochemical test evaluation

Following a 10–12 h fast, trained personnel collected blood and urine samples from participants upon enrollment. Serum was isolated from whole blood by centrifugation (1000 rpm, 15 min) and stored at -80 °C for subsequent analysis. Details regarding the standardized procedures employed can be found in the initial Hoveyzeh Cohort Study publication [33]. Fasting blood glucose was measured by the glucose oxidase method. TC, TG, HDL, BUN, creatinine and liver enzymes were determined by enzymatic kits (Pars Azmoon, Iran).

### Anthropometric measurement assessment

Trained personnel employed standardized procedures to measure participant height and weight with high precision. Height was assessed using a calibrated SECA 213 stadiometer to the nearest 0.1 cm, while weight was measured on a previously calibrated SECA 874 electronic scale accurate to 0.1 kg. BMI was also calculated as weight divided by height squared. The body shape index was calculated using the following formula [38]: ABSI = WC/[(BMI)ˆ (2/3) × (height)ˆ (1/2)]. The body roundness index was calculated using the following formula [39]: BRI = 365.2 − 365.5 ×$\sqrt {(1 - (((wc/2\pi )2)/{{[(0.5 \times height)]}^2}))}$. Also, we used from the following formulas for calculation of VAI [40]: Men: VAI = [WC/39.68 + (1.88 × body mass index[BMI])] × [triglycerides(TG)/1.03] × (1.31/HDL); women: VAI = [WC/36.58 + (1.89 × BMI)] × (TG/0.81) × (1.52/HDL). Both TG and HDL levels are expressed in mmol/L. TyG index was calculated with this formula [41]: Ln [fasting triglycerides (mg/dL) × fasting plasma glucose (mg/dL)/2]. Finally, we used from the following formulas for LAP index calculation: Female: LAP=(WC-58) × TG, Male: LAP= (WC-65) × TG.

### Physical activity assessment

Physical activity levels were assessed using the validated short version of the International Physical Activity Questionnaire (s-IPAQ) [42]. This 9-item tool quantifies time spent in moderate-to-intense physical activity sessions lasting at least 10 min, over the preceding seven days. Data analysis followed established IPAQ protocols [43], including conversion of hours to minutes, calculation of weekly frequency averages, and exclusion of participants with missing or ambiguous data (“do not know” or “refused”). Consistent with WHO guidelines, participants were categorized as inactive (< 150 min/week), moderately active (150–299 min/week), or highly active (≥ 300 min/week) based on their self-reported moderate-to-intense activity duration [44]. .

### Statistical analysis

Normality of variable distributions was confirmed using the Kolmogorov-Smirnov test. Continuous data were presented as mean ± standard deviation, while categorical data were expressed as percentages and counts. Differences in categorical variables across tertiles of the DII score were assessed with the chi-square test. One-way ANOVA examined significant mean differences in continuous variables across these same tertiles. Post-hoc comparisons using Bonferroni test revealed significant mean differences between DII tertile groups. Linear regression analyses, both crude and multivariable-adjusted, were employed to investigate the associations between the DII score and biochemical and anthropometric indices. The first model controlled for gender, education level, smoking, alcohol and age, while the second additionally included physical activity and calorie intake as covariates, and finally in the model 3, we additionally included waist circumference and BMI. The lowest DII tertile served as the reference category. Statistical significance in this study was set at *P* < 0.05, with borderline significance considered for *P* values of 0.05 to 0.07. Analyses were conducted using SPSS version 24 (SPSS Inc., Chicago, IL, USA).

## Results

### Participant flow

**Fig. 1:**
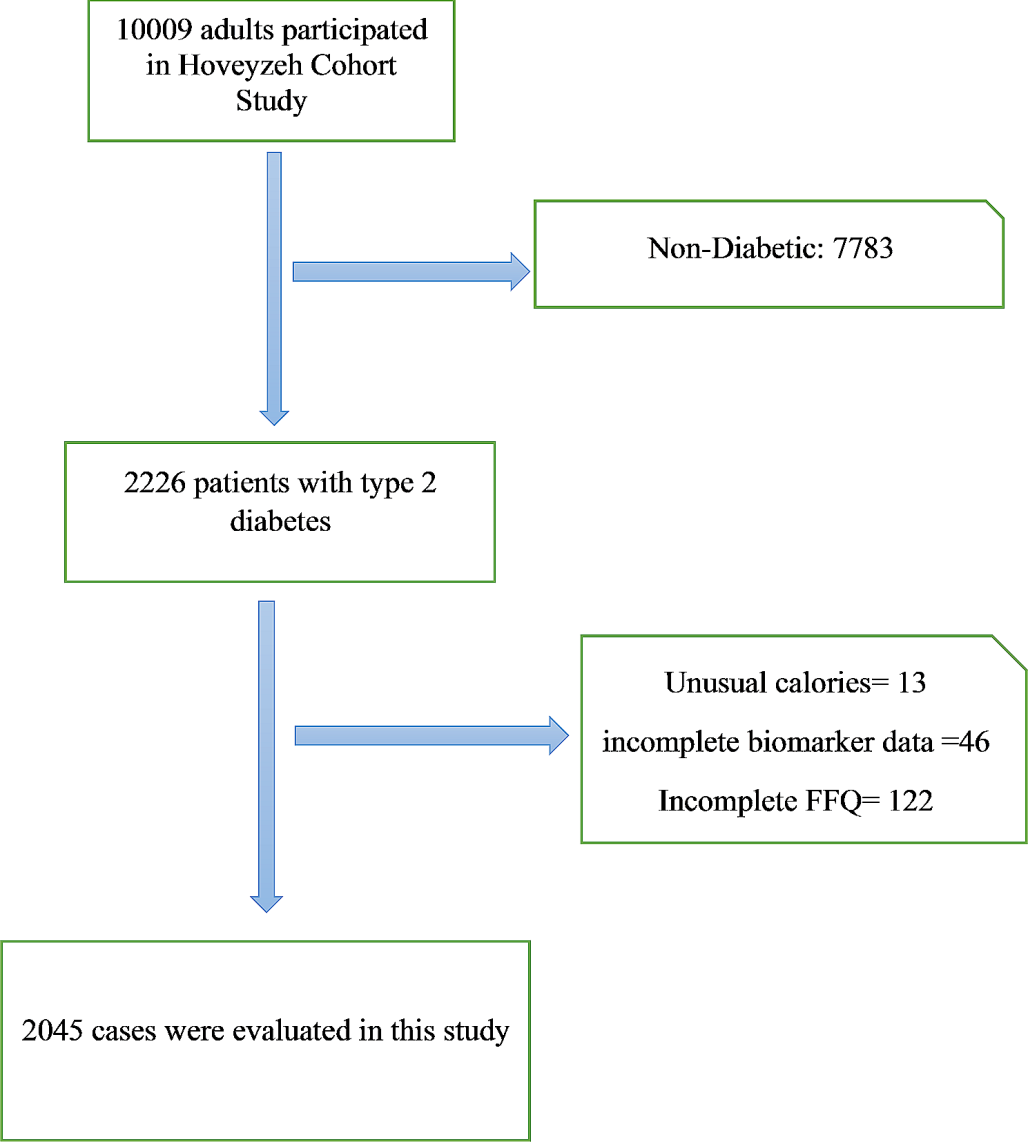
Study flowchart

Among 10,009 participants, finally 2045 patients we included in in the final analysis. The flowchart of patient selection process is summarized in Fig. 1.

### Baseline characteristics

Table 1 summarizes the baseline characteristics of participants across the DII score tertiles. A total of 2,045 patients were included in the final analysis. The participant population consisted of 36% males and 64% females. The mean age was 53.15 ± 8.93 years, with a significant difference observed across DII score tertiles (*p* < 0.001). We found significant differences (*p* < 0.05) in age, BMI, WC, weight, TyG, ABSI, LAP, systolic blood pressure (SBP), hemoglobin A1c (HbA1c), blood urea nitrogen (BUN), aspartate aminotransferase (AST), TG, and HDL levels between DII score tertiles. However, no significant differences were observed in other anthropometric and biochemical variables (*P* > 0.05). Additionally, 35% of patients reported using oral hypoglycemic agents and there weren’t any significant differences between DII tertiles in term of oral hypoglycemic agents’ usage (*P* = 0.72).

**Table 1:** Characteristics of the study population based on DII tertiles

Characteristic	Tertile 1	Tertile 2	Tertile 3	*P* value^a^
*N*	681	682	682	
Mean DII-density	-3.61 ± 0.87	-1.20 ± 0.61	1.13 ± 0.87	< 0.001
Age, years	50.51 ± 8.25	53.05 ± 8.89	55.88 ± 8.81	< 0.001
BMI, kg/m^2^	28.99 ± 5.15	29.30 ± 5.14	30.52 ± 5.19	< 0.001
WC, cm	101.98 ± 11.49	102.74 ± 11.33	104.51 ± 11.79	< 0.001
Weight, kg	74.91 ± 15.10	78.82 ± 14.27	83.50 ± 14.60	< 0.001
Height, cm	165.46 ± 8.75	164.10 ± 8.41	164.71 ± 8.20	0.49
WHR	0.98 ± 0.06	0.99 ± 0.062	0.995 ± 0.066	0.33
WHtR	0.63 ± 0.078	0.62 ± 0.079	0.64 ± 0.08	0.16
HC (cm)	102.74 ± 9.96	103.81 ± 10.23	105.85 ± 10.21	0.243
Oral hypoglycemic agents	240 (35.24%)	237 (34.8%)	239(35.04%)	0.72
BRI	6.33 ± 1.92	6.21 ± 1.93	6.40 ± 1.94	0.18
ABSI	0.83 ± 0.045	0.84 ± 0.047	0.85 ± 0.049	< 0.001
VAI	7.33 ± 5.99	7.74 ± 6.17	7.80 ± 6.65	0.32
TyG	9.45 ± 0.7	9.55 ± 0.71	9.60 ± 0.71	< 0.001
LAP	7872.51 ± 5909.93	8243.88 ± 5941.41	8946.74 ± 6225.69	0.004
Moderate activities, %				0.001
Yes	41.0	42.6	40.5	
No	59.0	57.4	59.5	
Smoking, %				0.32
Yes	12.7	11.4	13.2	
No	87.3	88.6	86.8	
Education level				0.41
Less than high school	22.3	23.5	25.7	
High school or above	77.7	76.5	74.3	
Gender				0.23
Male, number Female, number	249 432	258 424	230 452	
Use alcohol, number				
No	670	667	673	0.52
Yes	11	15	9	
FBS, mg/dl	179.73 ± 70.35	180.67 ± 73.25	173.68 ± 74.54	0.157
Systolic blood pressure, mmHg	128 ± 19	126 ± 16	124 ± 17	< 0.001
Diastolic blood pressure, mmHg	70 ± 14	71 ± 16	70 ± 13	0.243
Hemoglobin A1c, mmol/L	8.73 ± 1.3	9.12 ± 1.44	8.51 ± 1.12	< 0.001
BUN, mg/dl	13.38 ± 4.34	13.86 ± 4.97	14.76 ± 7.00	< 0.001
Cr, mg/dl	0.95 ± 0.25	0.98 ± 0.42	0.98 ± 0.44	0.319
ALT, U/L	17.78 ± 9.42	18.16 ± 10.77	17.31 ± 15.66	0.439
AST, U/L	22.84 ± 15.43	22.44 ± 13.56	19.27 ± 11.89	< 0.001
TG, mg/dl	205.37 ± 137.40	196.36 ± 137.36	184.09 ± 142.79	0.018
CHOL, mg/dL	193.98 ± 48.16	191.37 ± 44.71	195.16 ± 49.12	0.32
HDL, mg/dl	48.99 ± 11.84	48.27 ± 10.97	50.77 ± 12.30	< 0.001

^a^, *p*-values were determined using analysis of variance (ANOVA) test for continuous variables and Chi-square test for categorical variables. ^b^, Data are given as means, with standard division, ABSI, A body shape index; ALT, alanine amino transferase; AST, aspartate amino transferase; BMI, Body mass index; BRI, body roundness index; BUN, blood urea nitrogen; Cr, creatinine; CHOL, cholesterol; HDL, high density lipoprotein; HC, hip circumference; LAP, Lipid accumulation product; FBS, Fasting blood sugar; TG, triglyceride; VAI, visceral adiposity index; WHR, waist to hip ratio, WHtR, Waist-to-height ratio

### Dietary factors across different DII score tertiles

Table 2 presents dietary intakes of the study subjects categorized by DII score tertiles. We observed significant differences in carbohydrate and protein intake across DII score tertiles (*P* < 0.001). Compared to participants in the lower tertile, those in the higher DII tertile consumed significantly lower amounts of fiber, fruits, vegetables, legumes, fish and seafood, dairy products (*P* < 0.05), and exhibited a higher intake of red meat, processed meat, refined cereals, potatoes, and soft drinks (*P* < 0.05). Furthermore, significant differences (*P* < 0.001) were observed in the intake of some vitamins and minerals, with participants in the higher DII tertile consuming lower amounts of magnesium, vitamin A, vitamin C, vitamin E, and vitamin D compared to the lower tertile.

**Table 2:** Nutrient and food consumption according to tertiles of the dietary inflammatory index: among the patients with type 2 diabetes in the Hoveyzeh Survey

Variables	Tertile 1		Tertile 2		Tertile 3		*p*-value
	Mean	(95% CI)	Mean	(95% CI)	Mean	(95% CI)	
Energy (Kcal/day) Carbohydrates intake (% energy) ^b^	2632.4 54.3	(2433.7,2797.4) (53.1, 55.8)	2711.3 55.2	(2543.2,2844.5) (53.9,56.5)	2784.6 56.7	(2596.3.2944.6) (55.3, 58.4)	0.01 < 0.001
Protein intake (% energy)	15.2	(14.6, 15.5)	14.5	(14.2, 15.1)	13.8	(13.2,14.4)	< 0.001
Total fat intake (% energy)	31.3	(30.5, 31.8)	30.7	(29.6, 31.3)	30.5	(29.4, 30.9)	0.12
SFA (% energy)	11.6	(11.1, 12.2)	11.4	(10.8, 11.6)	11.3	(10.5, 11.5)	0.34
MUFA (% energy)	11.5	(10.9,11.8)	10.8	(10.3, 11.2)	10.6	(10.1,10.9)	0.109
PUFA (% energy)	7.8	(7.5, 8.4)	7.5	(7.1, 7.8)	7.4	(6.9, 7.7)	0.28
Fiber (g/day)	46.4	(43.9, 49.2)	30.2	(26.4, 34.3)	21.7	(17.4, 25.3)	< 0.001
Fruits (g/day)	304.6	(282.4, 335.7)	190.4	(160.4, 240.3)	112.2	(85.4, 15.70	< 0.001
Vegetables (g/day)	294.3	(273.4, 328.1)	188.42	(167.2, 319.6)	108.4	(82.5, 124.3)	< 0.001
Legumes (g/day)	61.3	(45.4, 76.9)	41.19	(33.5, 49.6)	23.6	(18.4, 29.4)	< 0.001
Fish and seafood (g/day)	21.5	(13.4, 29.3)	13.4	(9.3, 18.3)	8.4	(4.8,13.5)	< 0.001
Dairy products (g/day)	215.6	(190.4, 240.4)	167.3	(146.3, 184.5)	125.8	(103.4, 144.8)	< 0.001
Red meat and processed meat (g/day)	38.5	(31.6,43.7)	51.4	(44.34, 59.6)	64.3	(56.3, 68.4)	< 0.001
Eggs (g/day)	23.4	(16.5,31.7)	29.8	(26.4, 34.7)	34.2	(28.7, 37.6)	0.003
Refined cereals (g/day)	105.6	(94.3, 110.3)	128.4	(118.3,135.9)	145.9	(136.3, 150.4)	< 0.001
Potatoes (g/day)	8.3	(4.5, 12.7)	11.8	(8.5,15.9)	13.4	(9.2,17.32)	0.01
Soft drinks (mL/day)	74.6	(62.5,83.4)	112.4	(94.3, 128.3)	136.7	(120.4, 150.7)	< 0.001
Magnesium (mg/day)	584.6	(518.4, 647.3)	390.4	(312.7, 472.5)	267.3	(229.4,320.3)	< 0.001
Vitamin C (mg/day)	323.3	(264.2, 350.5)	243.2	(190.3,275.2)	185.4	(152.4,243.1)	< 0.001
Vitamin A (μg/day)	1532.4	(1276.1,1743.7	1143.3	(762.8, 1426.9)	719.4	(419.4,923.3)	< 0.001
Vitamin E (mg/day)	12.3	(8.4,15.3)	8.3	(5.6,11.9)	5.4	(3.7, 7.4)	< 0.001
Vitamin D (μg/day)	7.3	(5.8,8.4)	5.9	(4.8,6.7)	4.7	(3.4,7.1)	< 0.001

^a^*p*-values were determined using ANOVA test. ^b^ Data are given as means, with 95% CI in parentheses. SFA, saturated fatty acid; MUFA, Monounsaturated fat; PUFA, polyunsaturated fatty acids

### Association between DII score and biochemical variables

Table 3 presents the associations between DII score and various biochemical variables, including FBS, TG, total cholesterol, LDL cholesterol, HDL cholesterol, BUN, creatinine, ALT, AST, and the TyG index. No significant correlation was observed between DII score and FBS concentration in either the crude (*P* = 0.33) or fully adjusted model (*P* = 0.29).

**Table 3:** Results of linear regression for the association between DII and biochemical variables among the patients with T2DM

Variable	DII tertiles							*P* _trend_
	T1	T2			T3			
		β	SE	P1	β	SE	P1	
**FBS (mg/dl)**								
Crude	Ref	2.12	3.90	0.32	3.85	3.94	0.61	0.33
Model 1	Ref	1.26	3.99	0.46	2.90	3.95	0.75	0.48
Model 2	Ref	2.17	4.05	0.25	4.51	3.98	0.59	0.27
Model 3	Ref	3.28	4.04	0.08	7.06	3.94	0.41	0.29
**TG (mg/dl)**								
Crude	Ref	9.02	7.53	0.23	18.48	7.43	**0.014**	**0.02**
Model 1	Ref	9.59	7.54	0.21	19.46	7.62	**0.01**	**0.025**
Model 2	Ref	11.60	7.59	0.13	20.28	7.73	**0.008**	**0.005**
Model 3	Ref	10.68	7.58	0.17	19.62	7.76	**0.01**	**0.03**
**Cholesterol (mg/dl)**								
Crude	Ref	0.25	2.56	0.92	2.23	2.66	0.38	0.27
Model 1	Ref	1.04	2.33	0.52	1.63	2.59	0.68	0.59
Model 2	Ref	1.01	2.58	0.7	1.68	2.63	0.52	0.49
Model 3	Ref	0.78	2.48	0.77	1.79	2.69	0.49	0.48
**LDL (mg/dl)**								
Crude	Ref	0.91	2.1	0.65	1.49	2.36	0.46	0.45
Model 1	Ref	1.34	2.02	0.51	2.30	2.64	0.26	0.24
Model 2	Ref	1.56	2.01	0.43	2.74	2.05	0.18	0.19
Model 3	Ref	1.42	2.02	0.48	2.48	2.07	0.23	0.24
**HDL (mg/dl)**								
Crude	Ref	0.26	0.63	0.68	0.39	0.63	0.54	0.55
Model 1	Ref	0.59	0.61	0.33	1.002	0.62	0.107	0.107
Model 2	Ref	0.501	0.62	0.42	0.97	0.64	0.124	0.13
Model 3	Ref	0.36	0.61	0.56	0.73	0.63	0.24	0.25
**ALT**								
Crude	Ref	0.33	0.66	0.62	0.96	0.67	0.14	0.15
Model 1	Ref	0.21	0.66	0.75	0.89	0.67	0.18	0.21
Model 2	Ref	0.29	0.67	0.66	0.99	0.68	0.15	0.11
Model 3	Ref	0.41	0.67	0.53	1.26	0.68	**0.04**	**0.02**
**AST**								
Crude	Ref	0.23	0.74	0.75	1.20	0.74	0.106	0.11
Model 1	Ref	0.07	0.72	0.92	1.45	0.73	**0.04**	**0.02**
Model 2	Ref	0.15	0.73	0.84	1,58	0.82	**0.036**	**0.035**
Model 3	Ref	0.07	0.72	0.92	1.69	0.84	**0.03**	**0.033**
**BUN**								
Crude	Ref	0.31	0.3	0.32	0.73	1.12	0.15	0.11
Model 1	Ref	0.32	0.28	0.43	0.89	1.13	0.08	0.03
Model 2	Ref	0.11	0.29	0.34	0.92	1.13	**0.039**	**0.012**
Model 3	Ref	0.12	0.28	0.5	1.12	1.15	**0.023**	**0.01**
**Cr**								
Crude	Ref	0. 17	0. 23	0.23	0.3	0. 21	0.13	0.12
Model 1	Ref	0. 25	0. 22	0.17	0.43	0.27	0.06	0.05
Model 2	Ref	0. 13	0. 20	0.31	0.44	0.33	**0.03**	**0.02**
Model 3	Ref	0.19	0.24	0.25	0.51	0.35	**0.01**	**0.01**
**LAP**								
Crude	Ref	821.57	326.45	0.003	981.32	326.33	**0.012**	**0.012**
Model 1	Ref	705.62	325.99	0.002	965.33	329.66	**0.03**	**0.033**
Model 2	Ref	964.78	326.22	0.001	1107.60	332.26	**0.001**	**0.004**
**TyG**								
Crude	Ref	0.08	0.038	**0.022**	0.1	0.04	**0.038**	**0.008**
Model 1	Ref	0.09	0.04	**0.012**	0.12	0.042	**0.002**	**0.009**
Model 2	Ref	0.09	0.038	**0.014**	0.122	0.038	**0.002**	**0.002**
Model 3	Ref	0.1	0.04	**0.01**	0.13	0.041	**0.003**	**0.004**

Model 1, adjusted for gender, education level, smoking, alcohol and age, Model 2, Model 1 plus physical activity and calorie intake, Model 3, Model 1 and Model 2 plus waist circumference and BMI. ALT, alanine amino transferase; AST, aspartate amino transferase; BUN, blood urea nitrogen; Cr, creatinine; CHOL, cholesterol; FBS, Fasting blood sugar; HDL, high density lipoprotein; LAP, Lipid accumulation product; TG, triglyceride; TyG, triglyceride glucose index

Multivariate linear regression analysis revealed a positive association between DII score and TG concentration (P _trend_ < 0.05). Compared to participants in the first tertile, those in the third tertile of DII exhibited significantly higher TG levels in both the crude model (β = 18.48, SE = 7.43, *P* = 0.014) and the fully adjusted model (β = 19.62, SE = 7.76, *P* = 0.01).

Regarding liver enzymes, the present study found a positive correlation between DII score and both ALT and AST levels in the fully adjusted model (P _trend_ < 0.05). Furthermore, the fully adjusted model revealed a positive association between increased DII score and elevated serum creatinine and BUN levels (P _trend_ < 0.05).

Finally, a positive correlation was observed between DII score and both TyG index and LAP levels in both the crude and fully adjusted models (P _trend_ < 0.05). However, no significant association was found between DII score and other biochemical variables, including total cholesterol, LDL cholesterol, and HDL cholesterol (P _trend_ >0.05).

### Association between DII score and anthropometric indexes

Table 4 summarizes the associations between DII score and various anthropometric measurements. We observed a significant positive correlation (P_trend_ < 0.05) between DII score and ABSI, BRI, BMI, HC, WC and WHtR in both of the crude and full adjusted models (P _trend_ <0.05). Compared to participants in the first tertile, those in the third tertile of DII exhibited significantly higher values in the fully adjusted model for ABSI (β = 0.07, SE = 0.003, *P* = 0.04), BRI (β = 0.35, SE = 0.09, *P* < 0.001), BMI (β = 1.46, SE = 0.35, *P* < 0.001), HC (β = 2.36, SE = 0.54, *P* < 0.001), WC (β = 2.67, SE = 0.63, *P* < 0.001) and WHtR(β = 0.015, SE = 0.003, *P* < 0.001). However, we couldn’t find any significant correlation between DII score with VAI (P _trend_ =0.28) and WHR (P _trend_ >0.05).

**Table 4:** Results of multiple logistic regression for the association between DII and anthropometric indexes among the patients with T2DM

Variable	DII tertiles							*P* _trend_
	T1	T2			T3			
		β	SE	P1	β	SE	P1	
**ABSI**								
Crude	Ref	0.01	0.003	**< 0.001**	0.02	0.002	**< 0.001**	**0.01**
Model 1	Ref	0.04	0.002	0.06	0.05	0.003	0.07	**0.01**
Model 2	Ref	0.05	0.003	**0.02**	0.07	0.003	**0.04**	**0.02**
**BRI**								
Crude	Ref	0.18	0.11	0.08	0.41	0.1	**< 0.001**	**< 0.001**
Model 1	Ref	0.12	0.091	0.206	0.26	0.092	**0.005**	**0.005**
Model 2	Ref	0.17	0.091	0.06	0.35	0.093	**< 0.001**	**< 0.001**
**VAI**								
Crude	Ref	-0.05	0.34	0.87	-0.47	0.34	0.17	0.17
Model 1	Ref	0.001	0.35	0.99	-0.46	0.35	0.19	0.21
Model 2	Ref	-0.03	0.36	0.93	-0.51	0.45	0.27	0.28
**BMI**								
Crude	Ref	1.20	0.28	< 0.001	1.52	0.27	**< 0.001**	**< 0.001**
Model 1	Ref	1.21	0.27	< 0.001	1.72	0.29	**< 0.001**	**< 0.001**
Model 2	Ref	1.10	0.29	< 0.001	1.46	0.35	**< 0.001**	**< 0.001**
**HC**								
Crude	Ref	0.98	0.55	0.07	2.38	0.56	**< 0.001**	**< 0.001**
Model 1	Ref	0.81	0.53	0.12	1.78	0.53	**0.001**	**0.001**
Model 2	Ref	1.16	0.52	0.03	2.36	0.54	**< 0.001**	**< 0.001**
**WC**								
Crude	Ref	1.08	0.62	0.08	2.22	0.63	**< 0.001**	**< 0.001**
Model 1	Ref	0.93	0.61	0.13	1.88	0.62	**0.003**	**0.002**
Model 2	Ref	1.41	0.62	0.02	2.67	0.63	**< 0.001**	**< 0.001**
**WHR**								
Crude	Ref	0.001	0.003	0.66	0.0015	0.0035	0.58	0.61
Model 1	Ref	0.002	0.004	0.64	0.002	0.004	0.59	0.58
Model 2	Ref	0.003	0.003	0.39	0.004	0.004	0.24	0.25
**WHtR**								
Crude	Ref	0.008	0. 0.004	0.07	0.02	0. 005	**< 0.001**	**< 0.001**
Model 1	Ref	0. 005	0. 003	0.18	0.01	0.004	**0.002**	**0.004**
Model 2	Ref	0. 007	0. 003	0.049	0.015	0.003	**< 0.001**	**< 0.001**

Model 1, adjusted for gender, education level, smoking, alcohol and age, Model 2, Model 1 plus physical activity and calorie intake. ABSI, A body shape index; BMI, Body mass index; BRI, body roundness index; HC, hip circumference; VAI, visceral adiposity index; WHR, waist to hip ratio, WHtR, Waist-to-height ratio

## Discussion

To our knowledge, this is the first study to investigate the association between the DII and a range of anthropometric and biochemical indices in patients with T2DM. Our key findings revealed significant positive correlations between higher DII scores and several novel indices, including the TyG index, LAP, ABSI, BRI, and WHtR. Additionally, we observed significant associations between higher DII scores and several established variables, including TG, AST, ALT, BUN, BMI, WC, and HC. Notably, no significant associations were identified between DII score and other investigated variables.

Interestingly, while no significant association was observed between DII score and fasting blood sugar FBS in the present study, a positive correlation was identified with other biomarkers that elevate the risk of chronic diseases like chronic kidney disease (CKD) and non-alcoholic fatty liver disease (NAFLD) in patients with T2DM. This finding aligns with previous research emphasizing the connection between inflammation and diabetes [45–48]. Notably, King et al. conducted a cross-sectional study involving 4434 participants and reported an association between higher DII scores and elevated HbA1c levels [49]. Furthermore, Denova-Gutiérrez et al. reported a positive association between DII and T2DM risk in a separate population-based study conducted in Mexico City [50]. In their study, participants in the highest DII quartile exhibited a higher odds ratio for T2DM compared to those in the lowest quartile. In contrast, the present study population consisted primarily of patients with uncontrolled blood sugar levels, which may explain the lack of a significant association between DII and FBS concentration. It is possible that in patients with established T2DM and chronically elevated blood glucose, the association between DII and glycemic control becomes less direct. Instead, DII might influence factors more closely linked to disease progression, such as renal function (as indicated by BUN and creatinine levels) and anthropometric markers (e.g., BMI, WC) [51].

Our study identified a positive correlation between higher DII scores and TG levels. Additionally, we observed positive associations between DII and novel indices, such as the TyG index and LAP. These findings are noteworthy in the context of the well-established link between insulin resistance (IR) and both prediabetes/diabetes and cardiovascular disease (CVD) [52–55]. Recent research has emphasized the potential of novel anthropometric and biochemical indices as non-invasive and informative markers of IR [41, 56–59]. These indices, such as the VAI combining BMI and WC, LAP, TG and WC, and TyG index reflecting both TG and FBS, offer valuable tools for clinical assessment.

Our findings align with a growing body of evidence supporting the utility of these novel indices. Ahn et al., in a cross-sectional study involving 2,045 German participants, demonstrated that TyG and LAP provide a non-invasive and easily interpretable approach to identifying prediabetes/diabetes [60, 61]. Similarly, Shahavandi et al. observed an inverse correlation between adherence to plant-based dietary patterns and LAP levels in an Iranian population [62, 63]. These studies highlight the potential of dietary interventions to modulate inflammatory markers associated with metabolic health [64–66].

However, it is important to acknowledge some conflicting findings. Mirrafiei et al. reported a non-significant association between a meal-specific food-based DII and LAP or TyG levels in Iranian adults [67]. The relationship observed in the current study between DII and the indices related to IR and CVD can indicate the role of consumption of inflammatory diets in increasing the risk of CVD in diabetic patients. Inflammatory cytokines, like IL-1β and IL-6, can dampen the body’s ability to respond to insulin in fat, muscle, and liver. These cytokines are like pieces of a puzzle that explain how inflammation can lead to IR [68, 69]. Prolonged high blood sugar due to IR directly damages blood vessel cells (endothelium, smooth muscle) and immune cells (macrophages), triggering abnormal blood clotting and tissue breakdown. This interplay creates a breeding ground for atherosclerotic plaque formation. Additionally, excess reactive oxygen species and harmful protein-sugar molecules generated by hyperglycemia fuel chronic low-grade inflammation, further amplifying the risk of CVD [70, 71].

We also find a positive correlation between DII with ABSI, BRI and WHtR. Past research has explored WC, BAI, WHR, and WHtR for predicting metabolic risk, but these traditional anthropometric measures lack differentiation between fat and muscle mass [72–74]. To address this limitation, recent innovations like ABSI (incorporating WC, weight, and height) and BRI (using height and WC) have been developed to predict total and regional fat percentages and risk of cardio-metabolic disease more effectively [75–77]. Xu et al. in cross-sectional study with 17,000 Eastern-China adults found that BRI was significantly associated with high cardio-metabolic risk [31]. Recent studies investigated the effectiveness of two newly proposed indices, ABSI and BRI, in comparison to established measures like BMI and WC for predicting adverse health outcomes. Notably, these studies demonstrated that ABSI and BRI exhibited significantly stronger associations with abdominal fat accumulation, cardiometabolic risk factors, diabetes incidence, and premature mortality risks compared to BMI and WC [20, 78–80].

As mentioned, the present study was the first study that examined the relationship between the DII and novel cardio-metabolic risk indices. However, there were some limitations in the present study that should be considered. First, the study’s cross-sectional design limits the ability to establish causality. Second, dietary intake was assessed using a semi-quantitative food frequency questionnaire (FFQ), which may be subject to recall bias and misreporting of dietary intake. Third, While the analysis adjusted for several potential confounders, such as age, gender, education level, smoking, alcohol intake, physical activity, and calorie intake, there may be residual confounding from unmeasured variables (e.g., socioeconomic status, medication use, comorbidities) that could influence the observed associations.

## Conclusion

In conclusion, our findings reveal several noteworthy associations between the DII and various anthropometric and biochemical indices. Notably, we observed a positive correlation between higher DII scores and markers of insulin resistance, including triglyceride levels, TyG index, and LAP index. our study underscores the critical role of dietary inflammation in shaping cardio-metabolic health outcomes among individuals with T2DM. Future longitudinal studies incorporating more robust dietary assessment methods are warranted to validate our findings and elucidate the causal relationship between dietary inflammation and cardio-metabolic risk among patients with T2DM.

## Data Availability

The data that support the findings of this study are available from The Hoveyzeh Cohort Study (HCS), but restrictions apply to the availability of these data, which were used under license for the current study and so are not publicly available but is available with the corresponding author upon reasonable request.
